# The Efficacy of the Dyson Air Purifier in Improving Asthma Control: Protocol for a Single-Center, Investigator-Led, Randomized, Double-Blind, Placebo-Controlled Trial

**DOI:** 10.2196/28624

**Published:** 2021-07-27

**Authors:** Wei Chern Gavin Fong, Susan Grevatt, Stephen Potter, Tracey Tidbury, Latha Kadalayil, Kaisha Bennett, Maria Larsson, Frédéric Nicolas, Ramesh Kurukulaaratchy, Syed Hasan Arshad

**Affiliations:** 1 David Hide Asthma and Allergy Research Centre Newport United Kingdom; 2 School of Clinical and Experimental Sciences Faculty of Medicine University of Southampton Southampton United Kingdom; 3 Dyson Technology Limited Tetbury Hill Malmesbury Wiltshire United Kingdom

**Keywords:** air purifier, asthma, clinical trial, air pollution, allergens, respiratory function tests, bronchial provocation tests

## Abstract

**Background:**

Indoor air quality has been shown to influence asthma control and outcomes. Air purifiers and high-efficiency particulate air filtration devices can improve indoor air quality by reducing the indoor levels of air pollution and allergens. However, the influence of this improved indoor air quality on asthma control remains unclear; hence, randomized controlled trials are needed to further elucidate this phenomenon.

**Objective:**

This study aims to investigate the effect of reducing the levels of allergens and pollutants in the bedroom and living room through the use of Dyson air purifiers (Dyson Pure Cool) on asthma control.

**Methods:**

This is an 18-month long, investigator-led, randomized, double-blinded, placebo-controlled, single-center trial. Subjects will be randomized in a 1:1 ratio to active or placebo Dyson filters. The primary outcome is the change in the scores of Asthma Control Questionnaire 6 and Asthma-specific Quality of Life Questionnaire from baseline. Secondary outcomes include changes in lung function (forced expiratory volume in one second, forced expiratory volume in one second/forced vital capacity ratio, and midexpiratory flows), peak expiratory flow measurements, airway hyperresponsiveness (assessed by methacholine bronchial challenge), fractional exhaled nitric oxide, and indoor air pollutant levels. The sample size will be 50 subjects, and all subjects will have a confirmed diagnosis of mild persistent to moderate persistent asthma along with an Asthma Control Questionnaire 6 score of >1.5.

**Results:**

This study was approved by the West Midlands Research Ethics Committee (18/WM/0277). The study results will be published in peer-reviewed scientific journals; presented at relevant scientific conferences; and shared in plain English with participants in our newsletters, in our clinics, and via the David Hide Asthma and Allergy Research Centre website. Our trial began in September 2019 and is expected to end in August 2021.

**Conclusions:**

This is a double-blinded, placebo-controlled, randomized, investigator-led study to investigate the efficacy of a novel air purifier in improving asthma control in adults. The trial period of 18 months will facilitate the collection of robust data and will therefore generate clear signals. However, this extended trial duration may lead to patient withdrawal. Furthermore, this trial is conducted at a single center and in a location with a homogenous cohort of people, which may affect translatability. Nonetheless, it is hoped that the findings of this trial may help further inform clinicians regarding the utility of this novel device as an adjunct in asthma care.

**Trial Registration:**

ClinicalTrials.gov NCT04729530; https://clinicaltrials.gov/ct2/show/NCT04729530

**International Registered Report Identifier (IRRID):**

DERR1-10.2196/28624

## Introduction

### Background

The last few decades have been witnessing an ongoing *asthma epidemic*, whereby there is an increasing prevalence of asthma in the Western world and beyond [1]. The precise cause of this phenomenon is not fully understood, but it has coincided with changes in the quality of indoor air, which contains increased levels of allergens and pollutants [2]. The indoor environment contains other biological materials (such as microbiome and endotoxin) and pollutants (gases and particulate matter [PM]), which can adversely affect the development and morbidity of asthma [3]. Indoor pollutants also include smoke from cigarettes and wood, coal or gas fires, particulate materials associated with biofuel combustion, chemical vapors, gases including nitrogen dioxide (NO_2_), formaldehyde, and volatile organic compounds (VOCs). The latter may come from sources such as building products, cleaning agents, and paints. One such VOC is formaldehyde, which can irritate both the upper and lower respiratory tracts [4]. Small PM (PM2.5) is particularly damaging as it penetrates the small airways of the lungs and may even enter the bloodstream. Major indoor sources of NO_2_ and PM include gas stoves and cigarette smoke, but outdoor sources such as traffic and industrial pollution can also contaminate the indoor environment [5]. Among the indoor allergens, bedroom exposure to dust mite has been linked to worsening asthma symptoms and increased bronchial responsiveness [6]. In places where dust mites cannot thrive, allergens from cats, cockroaches, and Alternaria assume importance [7]. High indoor temperatures and humidity may increase the allergenic burden, particularly the proliferation of house dust mites and molds [7]. Therefore, modern living conditions are associated with a higher risk of allergen exposure, which causes an increase in the sensitization and symptoms of asthma.

It has also been suggested that exposure to pollutants can potentiate the effects of allergens [8]. Indeed, a combination of high levels of indoor pollution and allergens is related to the development and severity of asthma [9]. Allergens, microbiome, and pollutants can interact with each other to augment the immune response, further leading to harmful effects on the airways [10,11].

Thus, indoor air pollution (both chemical and biological) is a considerable environmental trigger for the acute exacerbation of asthma, which leads to increasing symptoms, emergency department visits, hospital admissions, and even mortality [3,9,12]. Therefore, maintaining a high air quality with lower levels of allergens and pollutants is important for improving the health of individuals with asthma and other respiratory diseases. A feasible and practical intervention that can reduce allergen and pollutant levels in indoor air should reduce morbidity and improve asthma control.

The National Institute of Health, United States, recently convened a workshop to examine the current status of the indoor environment in improving asthma control [9]. The workshop concluded that apart from the replacement of gas stoves with electric stoves and avoidance of indoor smoking, few methods are currently available for reducing indoor NO_2_ and possibly other pollutants. Technological improvements have been made in improving the efficacy of high-efficiency particulate air (HEPA) particle filtration devices that are designed to remove the targeted indoor air pollutants, such as fine PM (PM2.5) [13]. Some studies have demonstrated a positive effect of using an air cleaner with HEPA and carbon filters on asthma symptoms [14-16]. Although most studies using air purifiers have shown a reduction in gases and PM, the overall effect on asthma control remains uncertain. Allergen reduction intervention studies have reported inconsistent results [10]. Therefore, novel intervention strategies are needed to reduce both allergens and pollutants in the indoor environment to the extent that can improve asthma control and reduce airway inflammation and hyperresponsiveness. Recently, nocturnal temperature–controlled laminar airflow technology has been investigated for treating atopic asthma with some benefits [17]. It has been suggested that this strategy of reducing indoor exposure to allergens and pollutants might be cost-effective [18]. However, this result was not observed in a recent large randomized controlled trial investigating this technology [19].

### Objectives

The purpose of this study is to investigate the effects of reducing the levels of allergens and pollutants in the bedroom and living room, via a novel HEPA air purifier, on asthma control. Specifically, this study aims to investigate whether placing novel Dyson Pure Cool Towers [20], which have additional purification functions, in the bedroom and living room of subjects with asthma will improve their asthma control. The study hypothesizes that the addition of the Dyson air purifier to the standard treatment will improve asthma control, as assessed by the Juniper Asthma Control Questionnaire 6 (ACQ6) score [21], and improve the quality of life, as assessed by the Juniper Asthma-Specific Quality of Life Questionnaire (AQLQ) score [22], as a result of reducing indoor allergen and pollutant levels.

## Methods

### Study Design

This is an investigator-led, single-center, double-blinded, placebo-controlled, randomized controlled trial, with a 1:1 randomization ratio to an active or placebo filter. The David Hide Asthma and Allergy Research Centre (DHAARC), Isle of Wight National Health Service Trust, Isle of Wight, United Kingdom, will serve as the study site.

### Sample Size

As the trial will be using a novel intervention, the estimates of effect size are not available for a sample size calculation. However, other studies have shown that clinical response can be demonstrated using a HEPA filter with as few as 15 participants in each group [16]. Hence, we decided on a sample size of 25 in each group, which is likely to indicate whether the Dyson air purifier improves asthma control.

### Participants

We plan to recruit 50 participants from the David Asthma and Allergy Centre outpatient asthma clinics and primary care health centers, according to the inclusion and exclusion criteria (Textbox 1). All participants will be provided with oral and written information and adequate time to consider study enrollment. Written informed consent will be obtained by the research team.

**Inclusion and exclusion criteria.**

**Inclusion Criteria**

- Participants aged between 18 and 75 years with a confirmed diagnosis of mild persistent to moderate persistent asthma (defined as: British Thoracic Society guidelines [23] steps *regular preventer therapy* to *additional add-on therapies*).
- Juniper Asthma Control Questionnaire 6 score >1.5.
- Participants must be able to provide written informed consent.

**Exclusion Criteria**

- Participants with significant chronic respiratory diseases, such as chronic obstructive pulmonary disease or bronchiectasis.
- Participants with any severe disease (such as cardiovascular disease or dementia) because of which adherence to the study protocol may induce unjustified stress.
- Participants who are being treated with allergen-specific immunotherapy.
- Participants with a history of significant alcohol or drug abuse.
- Participants who are taking an investigational drug for asthma.
- Participants who were unwilling, unlikely, or unable to comply with the study protocol, as assessed by the study team members.
- Participants who were likely to be started on biological therapies for asthma (omalizumab, mepolizumab, reslizumab, benralizumab, and dupilumab) during the study period.
- Pregnancy.
- Participants already using air purifiers in their dwellings.
- Participants planning to shift from their houses during the study period.

### Intervention

The intervention studied will be the Dyson Pure Cool Tower, which will be installed in the living room and bedroom of study subjects. Dyson purifying fans sense particulates (PM10 and PM2.5) and gases (VOC and NO_2_) and capture pollution (HEPA filtration 99.95% PM0.1 and Tris-impregnated carbon granules to capture gases such as formaldehyde, benzene, and NO_2_). Then, they disperse purified air throughout the room through forward projection [20]. Study subjects can withdraw at any point and can continue to use their regular asthma medication.

### Primary Outcomes

The primary outcomes are changes in ACQ6 and AQLQ scores from baseline. A change in ACQ6 (≥0.5) score on a 6-point scale is considered the minimal important difference, whereas a change in AQLQ score of ≥0.5 on a 7-point scale is considered clinically important.

These questionnaires have been extensively validated and are commonly used in asthma research for measuring asthma control and quality of life.

These standardized questionnaires will be delivered over the telephone, by post, or in person during center visits.

### Secondary Outcomes

The secondary outcomes are as follows: (1) changes in airway responsiveness (assessed by methacholine bronchial challenge) from baseline, (2) changes in the indoor levels of pollutants (as measured by the Dyson purifier) from baseline, (3) changes in lung function assessed via spirometry (forced expiratory volume in one second [FEV_1_], FEV_1_/forced vital capacity [FVC] ratio, and midexpiratory flows) from baseline, (4) changes in peak expiratory flow (PEF) measurements from baseline, and (5) changes in airway inflammation (fractional exhaled nitric oxide levels [FeNO]) from baseline.

### Spirometry

Spirometry will follow the American Thoracic Society guidelines [24] to ensure validity and reproducibility. As per the guidelines, the highest of three FEV_1_ measurements within 5% of each other will be used. We will record FEV_1_, FVC, MEF, and PEF values in liters and their values percent predicted for age, height, sex, and ethnic origin. The forced expiratory ratio (FEV_1_/FVC) will also be calculated.

### Bronchial Hyperresponsiveness

Bronchial challenge will be performed using methacholine as the stimulant [25]. Methacholine will be prepared according to a prespecified protocol using a checklist that follows a specific standardized operating procedure consistent with the European Respiratory Society’s technical standards [26]. A safety checklist will be completed before each participant performs a bronchial challenge. The test will then be conducted according to the aforementioned standardized operating procedure consistent with that performed in previous studies at this center [27]. In total, 600 µg of Salbutamol will be prescribed on a standard hospital drug chart for all participants for administration after the bronchial challenge test. The test will be conducted by a doctor or a registered trained nurse (with immediate access to a doctor for additional support if needed).

A dosimeter system will be used (Jaeger APS [aerosol provocation system] Vyntus system, Carefusion). An initial inhalation of 0.9% saline will be followed 1 minute later by spirometry recording to obtain a baseline value. Baseline FEV_1_ will be required to be greater than 70% of the predicted value to proceed with the test. Subsequently, incremental methacholine doses from 1 mg to 32 mg will be administered. The dose causing a 20% decrease in FEV_1_ from the postsaline value will be interpolated and expressed as PD_20_ FEV_1_. A positive test is defined by PD_20_<2 µg. A continuous dose-response slope measure of bronchial hyperresponsiveness will also be estimated by the least-square regression of percentage change in FEV_1_ upon cumulative methacholine dose for each subject. The dose-response slope obtained will be transformed to satisfy normality and homoscedasticity. The results will be communicated to the participants’ general practitioners by written letters.

To perform spirometry or methacholine bronchial challenge, subjects will be required to be free from respiratory infection for 14 days, not taking oral steroids, not taken short-acting β2 agonists for 6 hours and long-acting β2 agonists for 12 hours, and abstained from caffeine intake for at least four hours. To exclude pregnancy before undergoing bronchial challenge, premenopausal female participants will be asked to provide a urine sample for pregnancy testing.

By way of safety precautions, participants will be followed up by telephone following each visit involving a bronchial challenge to check whether the participant is well and had no adverse reaction to the methacholine. All adverse reactions will be reported via the institutions’ local reporting system.

### FeNO Measurements

Measurement of FeNO will be performed in all patients using a standardized methodology [28]. This will be performed during each subject’s visit to the center.

### PEF Measurements

Measurements of PEF will be performed in all patients at regular intervals, according to the standardized technique. These data will be collected during spirometry. It will also be collected using either SMART peak flow technology [29] or the Wright Peak Flow meter (Clement Clarke International Ltd), based on patients’ preference.

### Additional Assessments

#### Additional Questionnaires

The standardized and extensively validated Juniper Rhinoconjunctivitis Quality of Life Questionnaire [30] will be administered. At baseline, the International Study of Asthma and Allergies in Childhood questionnaires [31] will be used to assess asthma symptoms, morbidity, medication requirements, and the presence of other allergic diseases. International Study of Asthma and Allergies in Childhood environmental questionnaires will be used at baseline to evaluate the home environment and exposure to allergens and irritants. An asthma history questionnaire (Multimedia Appendix 1) will also be used to assess asthma symptoms, morbidity, and medication requirements.

#### Physical Examination

Physical examination will include the assessment of vital signs, a general examination, and chest auscultation. Height and weight will also be recorded.

#### Dust Sampling

Dust samples will be taken from the mattress and carpet in the bedroom and living room (same rooms where the purifiers will be located). Sampling will be performed over an area of 1 meter square, which would be vacuumed for 2 minutes. The nozzle of the sampler will be wiped and thoroughly dried between the samples. The collected samples will be refrigerated and frozen at −20°C as soon as possible.

#### Blood Sampling

Blood samples will be collected at baseline and at the end of the study and stored at −80°C. The following samples will be collected:

1. 2×Green Top (BD Lithium Heparin 10 ml Plus–Bunzle Health care–VS367526)
2. 1×Lavender Top (Vacutainer blood sample tube K3EDTA 4 ml–KFK042/368860)
3. 1×Gold Top (Blood Sample Tube Serum Plastic 5 ml Gold with gel SST11–KFK114/367954).

#### Skin Prick Test

Skin prick tests will be performed by experienced nurses using standard techniques [32] and protocols. Antihistamines will be withheld (if safe to do so) for 72 hours before the procedure. A panel of 12 common allergens will be tested: house dust mite (*Dermatophagoides pteronyssinus* and farina), grass pollen mix, tree pollen mix, cat and dog epithelia, hamster, poultry feathers, *Alternaria alternata*, *Cladosporium herbarum*, Aspergillus, *Penicillium notatum* plus histamine (positive control), and physiological saline (negative control; extracts from ALK-Abello). An allergen skin test reaction with a mean wheal diameter of at least 3 mm greater than the negative control will be regarded as positive and the subject considered as atopic.

#### Indoor Air Pollution Data

Dyson will continuously sense the indoor pollutant data. The machines are equipped with air quality and environmental sensors on-board. This system also comprises sensors for measuring temperature, relative humidity, NO_2_, VOC, and PM. From the PM sensor PM2.5, PM10 is obtained. From the sum of the gaseous sensing and PM sensing, Dyson deduces an air quality index that is also displayed. When the product is connected to a local Wi-Fi, the data from these sensors are automatically sent to the Dyson cloud services [20]. These data will then be processed and shared with the study team for analysis.

### Randomization

Participants will be randomized to active (intervention) or placebo purifiers in a 1:1 ratio. The purifiers will be paired and randomized by Dyson using the random function in Excel (Microsoft), with the constraint that the two groups need to be equal. Each pair of machines will be allocated a number by Dyson. The study team will allocate a sequential DY number to the participants as they are recruited. Machine pairs will be allocated by number to the corresponding DY number.

### Blinding

Dyson will provide identical looking purifiers and placebo or active filters. Two purifiers will be wrapped and delivered to the subjects, where one machine will be installed in the bedroom and the other in the living room of the individual participant. Only the project manager will be aware of the serial numbers of the active or placebo filters. Participants, Dyson, and the study team will be blinded to participant allocation, but not the project manager. In addition, apart from knowing that the participants met the eligibility criteria, Dyson will be blinded to all other participant details. When filters or machines need to be changed, the project manager will be provided with the participant identifier (DY number). The project manager will then issue the study team a new machine with a matching filter status from the spare machines provided by Dyson, thus keeping the study team blinded.

### Data Management

Research participants will be seen at the DHAARC where all data will be gathered, processed, and stored securely for statistical analysis. Data management will follow a similar pattern used by a previous study [27]. An Access database (Microsoft), using the study number only for identification, will be set up. The current name and address will be entered into an Excel database. Study data will be stored in the Access database using the unique study number as the primary key, which will then be used to create separate analysis files in SPSS (IBM). The study number will link the subjects’ clinical information to other data and collected samples. Source data will be held securely on the Isle of Wight National Health Service local area network with limited access. The files used for analysis will not contain any personal identifiable information. Double data entry will be used for questionnaire data, whereas lung function data will be exported directly from the Carefusion Impulse Oscillometry machine (Carefusion). Range checks will be performed to ensure data quality.

Data will be collected and retained in accordance with the 2018 General Data Protection Regulation. All essential documents, including source documents, will be retained for a minimum period of 15 years following the end of the study. A *DO NOT DESTROY* label stating the time after which the documents can be destroyed will be placed on the inner cover of the records of trial participants. All study documents will be stored securely at the study site: the DHAARC at St Mary’s Hospital, Isle of Wight.

Anonymized data may be transferred to the University of Southampton, Faculty of Medicine, for further analysis. At the end of the trial, anonymized aggregated data will be shared with Dyson Technology Ltd for their review. We will establish a data-sharing agreement with both organizations. The sponsor will not make personal data, raw data, or any other results generated available to Dyson Technology Ltd or any third party, except for governance or audit by a regulatory authority.

### Data Analysis Plan

Data will be analyzed blinded with SPSS 26 (IBM Corporation), GraphPad Prism 9 (GraphPad Software), and Stata, version 16 (StataCorp).

Baseline descriptive statistics for both allocated groups will be presented and assessed. Normally distributed continuous variables will be presented as mean (SD) and assessed using two-tailed *t* tests. In case of deviations from normality, data will be suitably transformed before applying two-tailed *t* tests for comparison between the groups. Skewed data will be presented with median (IQR) and assessed using Mann-Whitney *U* tests. Categorical variables will be presented as percentages (frequency) and assessed using chi-square or Fisher exact tests.

The primary and secondary outcomes will be analyzed on an intention-to-treat basis.

The data on both primary and secondary outcomes from this study are longitudinal data with measurements taken at several time points. The starting point of asthma status and the response to intervention or placebo is expected to vary in a subject-specific manner over time within the group and between the groups. Therefore, the response data will be analyzed by fitting a multivariable linear mixed model (random intercept and random slope model) using the maximum likelihood estimation method with exposure groups as the fixed effect and time as the random effect. Such a model is well suited for accommodating missing data. Adjustments will also be made for the relevant baseline covariates. The assumptions of the model, normal distribution of residuals and constant variance, will be checked. Per-protocol analysis will also be conducted by using samples with no missing data. Separate-adjusted models will be built using relevant baseline covariates (eg, age, sex, and lung function).

### Patient and Public Involvement

Patients will not be formally involved in the study design. However, we sought the opinion of patients with asthma attending the clinic, and they had exceptional interest in obtaining evidence of the efficacy of Dyson air purifier in improving asthma control.

### Adverse Events

This study did not involve procedures with a greater than minimal risk. The social and psychological risks of talking to interviewers while completing questionnaires or documentation sheets are minimal. Skin prick testing may cause minimal discomfort and occasionally large localized swelling, but it rarely causes a systemic reaction [32]. Lung function tests and exhaled nitric oxide tests do not pose a significant risk of adverse effects. Bronchial challenge tests can cause wheezing and chest discomfort, but appropriate precautions will be taken to avoid this. Moreover, these symptoms can be treated with inhaled bronchodilators, which are routinely administered after the test. Very occasionally, nebulized bronchodilators may be required to treat more than mild wheezing and asthma symptoms that may have been provoked. Occasionally, patients with asthma can experience a severe episode of asthma following the test; hence, the test is always performed in the presence of a medically qualified and trained personnel and at well-equipped facilities for the immediate treatment of bronchospasm are at hand. The overall risk is similar to that of routine clinical care. If an adverse effect is reported at one of the regular contacts (center visit, home visit, and telephone interview) or during the study procedures, then all details will be recorded in the case report form and reported to the investigators if needed. Appropriate actions will be taken to eliminate or minimize the risk to the participant. In addition, participants will be provided with an emergency 24-hour telephone number to report any adverse effects. Any serious adverse events and adverse events will be tabulated. The study team will review safety data on an ongoing basis but specifically at their monthly meetings; moreover, all recorded adverse effects will be reviewed. The study team will be responsible for classifying, documenting, determining causality, and reporting. Adverse events will be recorded in accordance with Good Clinical Practice recommendations.

## Results

### Overview

**Figure 1:**
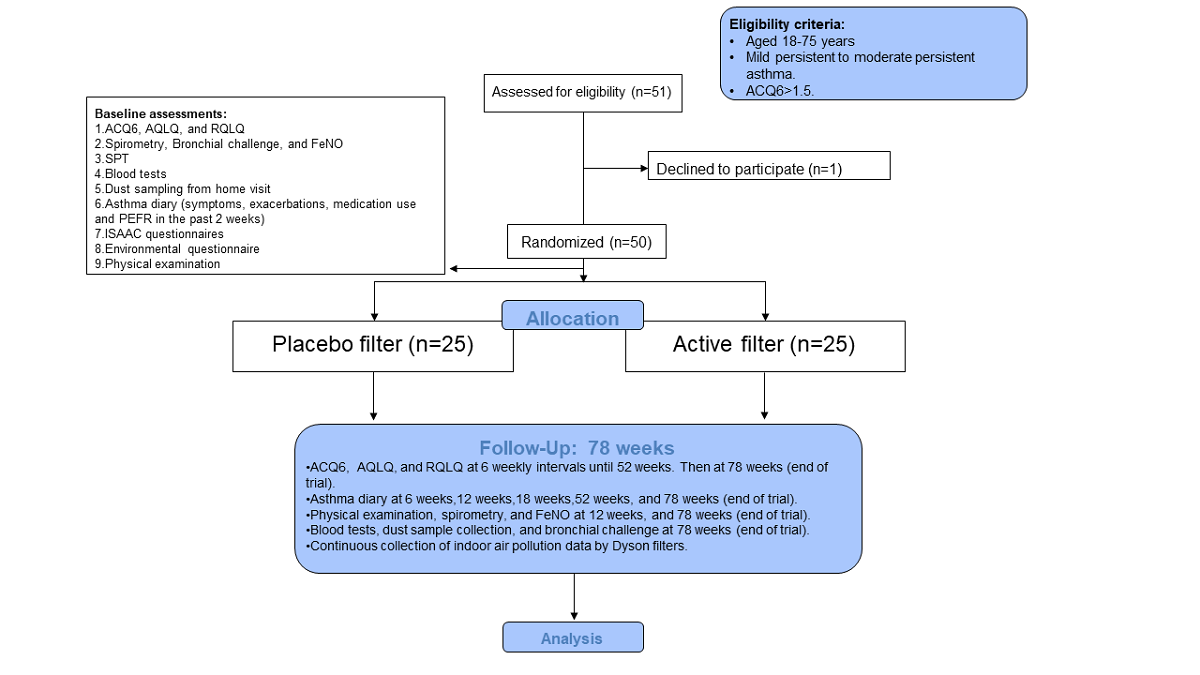
Overview and CONSORT (Consolidated Standards of Reporting Trials) diagram of the study. ACQ6: Asthma Control Questionnaire 6; AQLQ: Asthma-Specific Quality of Life Questionnaire; FeNO: fractional exhaled nitric oxide; ISAAC: International Study of Asthma and Allergies in Childhood. PEFR: Peak Expiratory Flow Reading; RQLQ: Rhinoconjunctivitis Quality of Life Questionnaire; SPT: skin prick testing.

The trial started in September 2019 and is expected to end in August 2021. A summary of the assessments and their intervals is presented in Table 1. An overview of the study design is shown in Figure 1.

**Table 1:** Trial assessment timepoints.

Study task or measurement performed	Screening or CV^a^1	HV^b^1, 0 week	TC^c^1, 6-7 weeks	CV2, 12-13 weeks	TC2, 18-19 weeks	TC3, 24-26 weeks	TC4, 30 weeks	TC5, 36 weeks	TC6, 42 weeks	TC7, 48 weeks	TC8, 52 weeks	CV3, 76-78 weeks	HV2, 76-78 weeks
Patient information and consent	✓^d^												
ISAAC^e^ questionnaires	✓												
ACQ6^f^	✓		✓	✓	✓	✓	✓	✓	✓	✓	✓	✓	
AQLQ^g^	✓		✓	✓	✓	✓	✓	✓	✓	✓	✓	✓	
RQLQ^h^	✓		✓	✓	✓	✓	✓	✓	✓	✓	✓	✓	
Asthma history questionnaires^i^	✓		✓	✓	✓	✓	✓	✓	✓	✓	✓	✓	
Peak expiratory flow	✓	✓	✓	✓	✓						✓	✓	
Physical examination	✓			✓								✓	
Skin prick test	✓												
Spirometry	✓			✓								✓	
Bronchial challenge methacholine)	✓											✓	
FeNO^j^	✓			✓								✓	
Dust sample collection		✓										✓	✓
Blood sample	✓											✓	
Installation of air purifiers		✓											
Indoor air pollution data by Dyson purifiers		✓	✓	✓	✓	✓	✓	✓	✓	✓	✓	✓	✓
Return of air purifiers													✓
Letter to general practitioner	✓											✓	

^a^CV: center visit.

^b^HV: home visit.

^c^TC: telephone call.

^d^Task or measurement is performed.

^e^ISAAC: International Study of Asthma and Allergies in Childhood.

^f^ACQ6: Asthma Control Questionnaire 6.

^g^AQLQ: Asthma-Specific Quality of Life Questionnaire.

^h^RQLQ: Rhinoconjunctivitis Quality of Life Questionnaire.

^i^Asthma history questionnaire includes information on symptoms, medication use, and exacerbations in the past 2 weeks.

^j^FeNO: fractional exhaled nitric oxide.

### Ethics and Confidentiality

The study protocol was written in accordance with the Standard Protocol Items: Recommendations for Interventional Trials statement [33], and the trial will be reported according to the guidelines of the CONSORT (Consolidated Standards of Reporting Trials) statement [34].

The West Midlands Research Ethics Committee (REC; REC Reference, 18/WM/0277) and the local research and development department approved and reviewed this study. The study will be conducted in accordance with the principles of Good Clinical Practice. Any amendments to the protocol will be submitted to the REC and local research and development for review and approval.

All records, reports, and forms will be anonymized and identified in a manner that ensures participant confidentiality. Records will also be stored in a secure area with limited access. Clinical information will not be released without the written permission of the participant, except as necessary for monitoring and auditing by the sponsor, its designee, or the REC or as required by law. The investigators and study site staff involved in this study may not disclose or use, for any purpose other than the performance of the study, any data; record; or other unpublished, confidential information disclosed to the individuals in this study. Previous written agreement from the sponsor or its designee must be obtained for the disclosure of any said confidential information to other parties.

### Dissemination of Research Findings

The chief investigator will serve as the custodian of the data arising from this study, and the sponsor will be the owner of the data. The study findings will be used for publication in peer-reviewed scientific journals and presentations in scientific meetings. Summaries of the results will also be made available to investigators for dissemination within their clinics. The findings will also be shared in plain English with participants in our newsletters and via the DHAARC website.

## Discussion

This protocol outlines a double-blinded, placebo-controlled, randomized, investigator-led study designed to investigate the efficacy of a novel air purifier in improving asthma control in adults. The trial period of 18 months will facilitate the collection of robust data and will therefore generate clear signals. However, this extended trial duration may lead to patient withdrawal and adherence challenges. Furthermore, this trial is conducted at a single center and is conducted in a location with a homogenous cohort of people, which may affect translatability. Nonetheless, it is hoped that the findings of this trial may help further inform clinicians regarding the utility of this novel device as an adjunct in future asthma care. In addition, the comprehensive air pollution data captured by the device along with the repeated characterization of subjects via subjective and objective measures will add to the understanding of the influence of indoor air pollution on asthma control.

## Appendix

Multimedia Appendix 1 Asthma history questionnaire.

## Abbreviations

ACQ6: Asthma Control Questionnaire 6
APS: aerosol provocation system
AQLQ: Asthma-Specific Quality of Life Questionnaire
CONSORT: Consolidated Standards of Reporting Trials
DHAARC: David Hide Asthma and Allergy Research Centre
FEV1: forced expiratory volume in one second
FVC: forced vital capacity
FeNO: fractional exhaled nitric oxide levels
HEPA: high-efficiency particulate air
NO_2_: nitrogen dioxide
PEF: peak expiratory flow
REC: Research Ethics Committee
PM: particulate matter
VOC: volatile organic compound
