# Supplementary material for: The Efficacy of the Dyson Air Purifier in Improving Asthma Control: Protocol for a Single-Center, Investigator-Led, Randomized, Double-Blind, Placebo-Controlled Trial
Source: JMIR Res Protoc. 2021 Jul 27;10(7):e28624. doi: 10.2196/28624 (PMC8367098; doi:10.2196/28624)
Supplement: Multimedia Appendix 1 [file resprot_v10i7e28624_app1.pdf]

The David Hide Asthma and Allergy Research Centre
Dyson Air Purifier in Asthma Study

Study No: | | | | |

<u>Asthma History Questions:</u> **Date of Visit: ……………………………**

Not Known

1. Number of GP Visits for Asthma in past 3 months? …………………………… ☐
2. Number of OCS (Oral Steroid) courses in past 3 months? …………………………… ☐
3. Number of ED Asthma Visits in past 3 months? …………………………… ☐
4. Number of Asthma Hospital Admissions in past 3 months? …………………………… ☐
5. If Admitted to Hospital

   a) how many days did the admission last? …………………………… ☐

   b) were admitted to ICU (Intensive Care) at any point?

Yes	No	Not Known

6. Asthma Medications currently being taken

Name of Drug	Strength/Dose	Dosing Frequency	Number of prescriptions picked up past 3 months	Number of doses per prescription	Additional Comments
FOR EXAMPLE *Fostair*	*100/6*	*2 puffs twice daily*	*4*	*Admits to occasionally forgetting evening doses*	

7. Any Asthma Medications discontinued or started in the past 3 months?

Name of Drug	Strength/Dose	Dosing Frequency	Additional Comments e.g reason for stopping or starting
